# The impact of COVID-19 on access to dental care for people with disabilities: a global survey during the COVID-19 first wave lockdown

**DOI:** 10.4317/medoral.24742

**Published:** 2021-08-19

**Authors:** Caoimhin Mac Giolla Phadraig, Maria T van Harten, Márcio Diniz-Freitas, Jacobo Limeres Posse, Denise Faulks, Alison Dougall, Pedro Diz Dios, Blánaid Daly

**Affiliations:** 1 School of Dental Science, Trinity College Dublin, Dublin 2, Ireland; 2 Department of Child and Public Dental Health, Dublin Dental University Hospital, Trinity College Dublin, Dublin 2, Ireland; 3 Medical-Surgical Dentistry Research Group (OMEQUI), Health Research Institute of Santiago de Compostela (IDIS), University of Santiago de Compostela (USC), Spain; 4 CHU Clermont-Ferrand, Service d’Odontologie, F-63003 Clermont-Ferrand, France; 5 Université Clermont Auvergne, CROC, F-63000 Clermont-Ferrand, France

## Abstract

**Background:**

It is unclear what immediate impact the COVID-19 pandemic has had on delivery of oral healthcare to people with disabilities worldwide. Aim: To report the international impact of COVID-19 lockdown on oral healthcare provision for people with disabilities before, during and after the first lockdown (March to July 2020).

**Material and Methods:**

Cross-sectional online self-administered survey of dentists who treat people with disabilities completed 10th to 31st of July 2020. Responses allowed comparison from before, during and immediately after the first wave lockdowns of the COVID-19 pandemic. Data were analysed using McNemar’s test to compare reported practice before to during lockdown, and before to after lockdown.

**Results:**

Four-hundred-thirty-six respondents from across global regions reported a significant reduction from before to during and from before to after lockdown regarding: the proportion of dentists treating people with all types of disability (*p* <0.001) and the number of patients with disabilities seen per week (*p*<0.0001). The proportion reporting no availability of any pharmacological supports rose from 22% pre-lockdown to 61% during lockdown (*p* < 0.001) and a persistent 44% after lockdown (*p* < 0.001). An increase in teledentistry was observed.

**Conclusions:**

During the first COVID-19 lockdown, there was a significant negative impact on the delivery of dental care to people with disabilities. Oral healthcare access was significantly restricted for people with disabilities with access to sedation and general anaesthesia particularly affected. There is now an increased need to ensure that no-one is left behind in new and existing services as they emerge post-pandemic.

** Key words:**COVID-19, disability, dental care, access.

## Introduction

On the 5th of January 2020, the WHO published its first report of an outbreak of pneumonia of unknown origin in Wuhan, China that would come to be known as the COVID-19 pandemic. The pandemic was caused by a novel coronavirus termed severe acute respiratory syndrome coronavirus 2 (SARS-CoV-2) (1). Within two weeks of this initial report, cases were observed in Thailand, Japan and Korea. By mid-March, cases had been diagnosed across every major region of the globe, sparking a range of social and public health measures aimed at “flattening the (epi-) curve” (2).

While specific strategies varied from country to country, and noTable exceptions arose, the general aim was to prevent the introduction of SARS-CoV-2, control its spread and reduce the burden on the health system (2). Specific examples of such measures included cancellation of public events, social distancing, travel restrictions, case identification, contact tracing and related measures, and risk communication strategies (3,4). National strategies that featured restricting movement and community interactions came to be known as a “lockdown”. Following the first Lockdown in Hubei province in January 2020 (5), many countries followed suit with full or partial lockdowns to manage the first wave of the pandemic (6). While the exact meaning varied from country to country, lockdown commonly consisted of closure of non-essential workplaces, schools, colleges, hospitality, recreation facilities and places of worship, while essential services in healthcare, transport, food services and supply chains remained open (7).

As the lockdowns of the first wave of the pandemic set in, dental professionals developed and shared strategies for the safe delivery of dental care, in recognition that dental settings have unique characteristics that warrant specific considerations: multiple people within close proximity, confined spaces, production of aerosols and secretions, and the unavoidable nature of urgent care (8,9). Together, these conditions generated a sense of increased risk among dental professionals and their patients. In the understandable absence of strong guidance, the exact measures adopted varied, based in turns on National Guidance (e.g. the Scottish Dental Clinical Effectiveness Programme), individual discretion or the advice of representative organisations (10). Specific measures were recommended by the likes of the Cochrane Oral Health CoDer Group and others (9), which included: complete closure of general dental practices and establishment of emergency hubs nationwide; limiting treatment to emergency care when not manageable with advice, analgesics and antimicrobials; remote consultations; changes to personal protective equipment; staff and patient risk assessment protocols; avoiding aerosol generating procedures; infection prevention and control measures; and social distancing within practices. Arrangements in many countries were ad-hoc, planned outside of other medical care and failed to provide clear guidance for patients on accessing emergency dental care.

Failures in nationally coordinated responses meant that access to dental care was severely restricted. Opinion varied on the impact of such restrictions with some arguing that oral health services worldwide are so poor and oral disease already so prevalent, little impact would be felt at a global public health level (11). Other commentators feared that social inequalities in oral health would be exacerbated due to the differential impact of COVID and COVID restricted access to healthcare across socio-economic gradients (12). The consequences of the protective measures listed above were unknown, though early reports indicated that patients were voting to defer basic dental treatment (13).

According to the WHO and United Nations, the COVID-19 pandemic brought a range of additional burdens for people with disabilities, who constitute approximately 15% of the world population. People with disabilities were deemed at increased risk of and from COVID 19 due to a range of specific issues, such as relying on close contact for personal care, difficulty with basic hand hygiene, communication challenges, difficulty accessing appropriately formatted information, co-morbid health conditions, underlying socio-economic disparities associated with disability, risk from residential setting, stigma and isolation and reduced access to essential support and healthcare services. On top of the general restriction to healthcare faced by all members of the public, concerns were raised that the additional efforts to isolate and protect people with disabilities would negatively impact their access to healthcare, thereby compounding pre-existing inequalities. This fear has since manifested for people with physical and neurodevelopmental impairments (14,15).

At the time of first wave of lockdowns, similar concerns were raised regarding the potential for disproportionate impact on access to oral healthcare for people with disabilities internationally. Up to that point people with disabilities were already experiencing inequity in interactions with oral healthcare services (16). They often experienced ineffective and inappropriate care leading to tooth loss, edentulism and periodontal (gum) disease (17). Often, people with disabilities found it difficult to access appropriate oral healthcare. Barriers abounded around physical access and treatment was often only sought in emergencies, rather than for preventive care. Attitudes among oral healthcare providers were also wanting and choice for people with disabilities was absent (18).

Bearing this preexisting inequity in mind, lockdown led to additional barriers: anecdotal evidence emerged of unmet urgent dental needs among people with disabilities, declining access to dental general anesthesia and sedation services, and mass repurposing of public oral health professionals (10,19,20). It seemed that people with disabilities were facing novel barriers to oral health and access to appropriate oral care, superimposed upon existing barriers to care. However, no data existed to verify or quantify these observations. This study therefore explores the international impact of COVID-19 lockdown on oral health provision for people with disabilities by surveying special care dentists regarding their practice before, during and after the first lockdown circa March to July 2020.

## Material and Methods

- Design

This study adopted a cross-sectional online self-administered survey design to answer the question “Is there a difference in the international delivery of dentistry for people with disabilities between before, during and after the first wave COVID 19-related lockdown?” Ethical approval was received from the Research Ethics Committee of the University of Santiago de Compostela (Spain) (reference code USC-10/2020).

- Participant Recruitment

The survey was open to all dentists around the world, who provide Special Care Dentistry (SCD), the arm of dentistry that focuses on the oral care of people with a wide range of disabilities and special healthcare needs. Opportunistic sampling was applied. Global and regional networks of special care dentists were targeted to ensure global engagement. Social media were used to raise awareness among potential participants. Snowballing was sought, whereby sharing of the survey URL with professional colleagues working in SCD was encouraged.

- Survey Development

In the absence of a pre-existing tool or suiTable instrument validated for such an unprecedented situation, an initial pool of questions were drafted by the lead author and two experts in the field of SCD. These questions were reviewed by an international expert panel of six specialists in SCD and a statistician. Questions were selected from this pool to answer the research questions and additional items added to ensure that areas considered important were covered. The survey in its final form consisted of four sections: 1. Demographics and professional status; 2. Experience of restrictions on dental practice during the first international COVID-19 pandemic wave; 3. Changes in practice over time related to COVID-19 restrictions; and 4. Expectations for the future.

This study explores section 3 from this list, with other sections published in another source (21). Responses to the questions explored in the current study required participants to reflect on features of their practice immediately before lockdown, at the height of lockdown, and then at the time of their completion of this survey, which was open between the 10th and 31st of July 2020, a time when restrictions were generally eased.

- Data Collection and Analysis

Questions were entered into Google Forms for electronic distribution and data collection. Data was exported to Microsoft Excel for cleaning and manipulation of variables for further analysis. Analyses were carried out using SPSS v.26. Demographic data were described using counts and percentages. Responses to questions from the section ‘Changes in practice over time related to COVID-19 restrictions’ that were “check all that apply”-styled were converted to dichotomous “yes/no” responses to each item on the list using dummy variables.

Data were analysed using McNemar’s test where dichotomous observations could be matched by participant to compare perceptions about practice before lockdown to during lockdown, and before lockdown to the time of survey completion. McNemar’s test, using the binomial distribution, is appropriate for non-parametric analysis of 2x2 cross-tabulations for paired data. Where a response about one time period could not be paired with the individual’s other time period, these observations were excluded from any analysis. Where analysis of larger contingency Tables was required because of categorical variables having more than two levels, McNewmar-Bowker’s test was used to investigate symmetry of pre- and post-COVID-19 lockdown responses paired by individual (22).

## Results

- Demographics

Four hundred and thirty six dentists from all world regions responded (Table 1): Forty percent were practising in Europe, 23% in Latin America and the Caribbean, 21% in North America. Seventy-one percent of participants were female and 55% of participants were between the ages of 30 and 50 years, with 53% having been practicing dentistry for over 20 years. Forty-five percent practiced in a publicly-funded dental clinic, 29% in a privately-funded clinic, and 24% practiced in both sectors.

- Type of disability and numbers accessing dental care

The most common groups of patients reported by participants as being seen before lockdown were those with intellectual/developmental disabilities (86%), medically compromised patients (81%) and those with physical disabilities (78%) (Table 2). Other groups, such as patients with neurological disorders and sensory disabilities, the frail and elderly, and anxious or phobic patients were seen by fewer participants. During lockdown, the number of participants treating patients from each of these disability groups dropped (*p*<0.001), and this continued to the time of their participation in this survey (i.e. when restrictions had eased or were easing) (*p*<0.001).

**Table 1:**
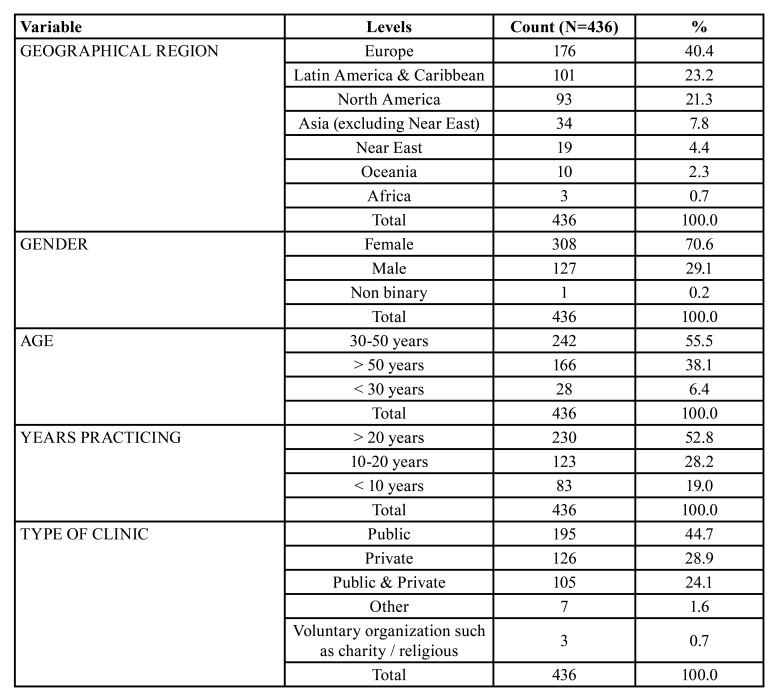
Demographics.

Likewise, the numbers of dentists seeing many patients with disabilities dropped during lockdown (*p*<0.001) and had still not recovered to pre-lockdown levels at the time of the survey (*p*<0.001).

- Changes in the type of dentistry provided

The most common types of care provided to patients before lockdown were SCD, paediatric dentistry, and general dentistry with 80%, 56%, and 51% of study participants saying they were delivering these (Table 3). All other types of dentistry were delivered to a lesser extent. During lockdown, the number of participants delivering all types of care dropped except general dentistry (*p*<0.05). At the time of their participation in this survey, participants were still reporting reduced delivery of SCD, paediatric dentistry, periodontics and prosthodontics compared to pre-lockdown (*p*<0.05); while other types of dentistry including general, oral surgery, implantology, endodontics, orthodontics, and oral medicine reverted to baseline levels (*p*>0.05).

- Changes in the availability of pharmacological support

The most common pharmacological supports available to participants were for general anesthesia, relative analgesia/nitrous oxide sedation, and oral conscious sedation, with 69%, 66%, and 53%, respectively reporting access before lockdown restrictions were put in place (Table 4). Intravenous conscious sedation and/or deep sedation were accessible to fewer participants, while 22% reported no access to any pharmacological supports at all. During lockdown, the number of participants accessing all forms of pharmacological supports was significantly reduced compared to pre-lockdown (*p*<0.01), with the number reporting no availability of any pharmacological supports rising from 22% to 61% during lockdown (*p* < 0.001) and a persistent 44% after lockdown (*p* < 0.001). At the time of their participation in this survey, participants were still reporting reduced access compared to pre-lockdown (*p*<0.05).

**Table 2:**
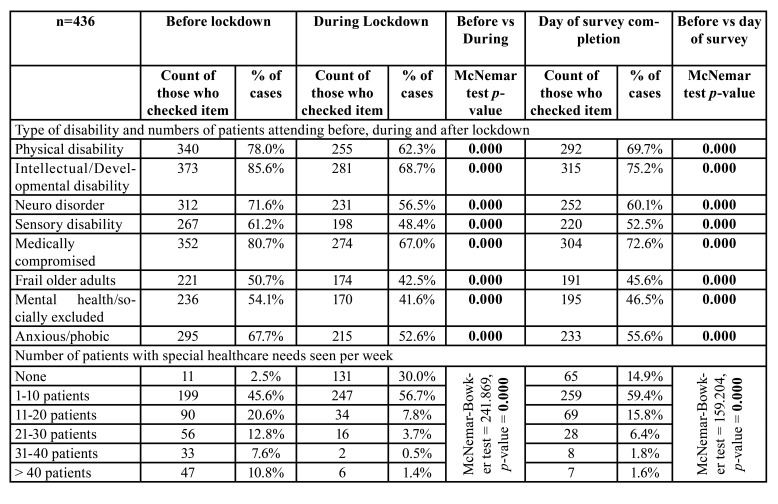
Change in category of disability types and numbers accessing dental care.

**Table 3:**
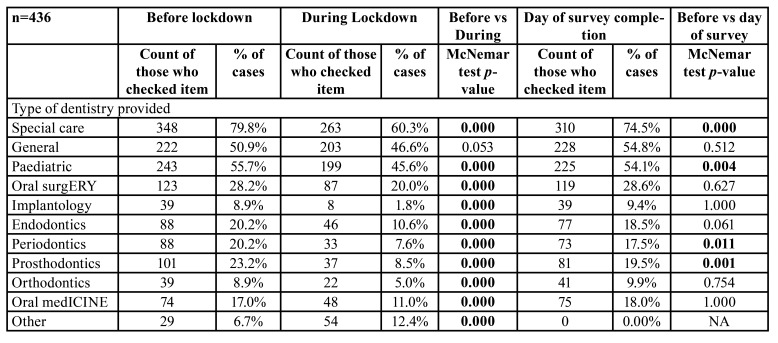
Change in the type of dental care provided.

- Changes in use of digital services for communication

Also, in Table 4, the most popular means of communication with patients utilised by participants before lockdown were telephone and email which were used by 84% and 50% of respondents respectively (Table 4). Less common means were social media, videoconferencing and teleconsultation. During lockdown, the number of participants using email, social media, videoconferencing and teleconsultation rose (*p*<0.05). At the time of their participation in this survey, participants were still reporting usage of these four electronic means at higher levels than before lockdown (*p*<0.05).

**Table 4:**
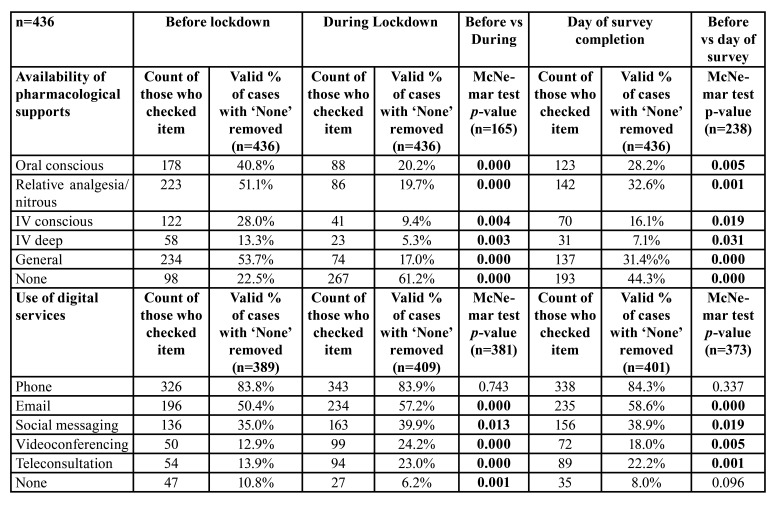
Change in availability of pharmacological supports and use of digital services.

## Discussion

This study demonstrates the significant impact of COVID-19 on the delivery of oral health care to people with disability, that were already insufficient, during the first wave of COVID-19 lockdown. Respondents in this study were dentists who provide Special Care Dentistry to a broad range of people with physical, neurodevelopmental, neurological, sensory, medical, age-related, social, and mental health related impairments. Respondents came from across the globe, had a range of experience, and represented a broad range of healthcare settings, coming from public, private and mixed practice settings. They reported three important changes to oral healthcare for people with disabilities during lockdown, most of which indicate potentially persistent decline in the quality and quantity of oral healthcare, and further reduction in access to appropriate care for people with disabilities globally.

Firstly, respondents reported a decline in the provision of all dental care for people with disabilities during lockdown. Reductions were observed across disability types, so no one type of disability appeared to have been disproportionately excluded from accessing dental care, slight variations aside. The number of people with special healthcare needs seen by dentists declined dramatically during lockdown and beyond, with persistent increase in the number of respondents seeing fewer than ten people with disabilities a week and a dramatic reduction in those seeing more than this. This finding is deeply concerning. It is not known what impact this reduction has had on the lived experience of people with disabilities. Within this population, many are unable to express or communicate pain conventionally. These people often present with acute, end stage disease and severe infection, as their care givers have difficulty identifying earlier signs and symptoms of dental problems. These same patients are those that are least likely to be able to access or to cooperate with treatment in the mainstream dental environment. People with disabilities faced immense barriers to accessing appropriate oral healthcare services even before the COVID-19 pandemic (16,23,24). Often oral health services failed to meet their needs and left them with poor outcomes such as total tooth loss, uncontrolled tooth decay and gum disease (17). This study finds that the COVID-19 pandemic has had an additional impact on the spectrum of people with disabilities’ ability to access oral healthcare services that were already insufficient.

Secondly, dentists reported a significant reduction in access to pharmacological supports for patients with disabilities. At the height of lockdown, the proportion of respondents with no access to any of these adjuncts tripled compared to before lockdown, and those who had these available saw ongoing reduced access to most forms of sedation and particularly general anaesthesia. These changes persisted beyond lifting of restrictions, with only oral sedation, a limited option, approximating pre-COVID availability. This is especially important given the pre-existing limited global access and the high proportion of people with disabilities who require pharmacological support in order to access safe and appropriate dental care, particularly those with profound disabilities, behavioural and communication issues (25).

Thirdly, and rather more positively, respondents reported an increase in the use of digital services for communication with patients, particularly using tele-consultations and video conferencing. These changes maintained access to a form of oral healthcare while respecting isolation and distancing. Again, these changes persisted once initial lockdown restrictions began to lift. This change is a significant positive for people with disabilities. While the dental profession has been slow to adopt this technology, the benefits from teledentistry have long been espoused and it is people with disabilities who have most to gain. Teledentistry is a seemingly cost effective means of overcoming barriers due to geographic distance between patients and particularly specialist care providers, who usually condense in large urban areas. This approach also surmounts barriers surrounding transportation and related out of pocket and opportunity cost, while offering accurate diagnosis and opportunity for definitive or intermediate management of many orofacial conditions across an expanded network of care providers (26-29). Although teledentistry does not and cannot replace clinical intervention, particularly in situations where a tooth needs to be opened or an abscess drained, it is invaluable in facilitating communication with specialist healthcare providers and subsequent access to appropriate care that takes into account the patient’s special circumstances and needs.

This study should be considered in context of its strengths and limitations. The strength of this study is that it is the first to quantify the impact of COVID-19 on delivery of dental care to people with disabilities. Additionally, the study involves a large international sample, giving a global perspective, albeit with limited representation from some world regions. However, there are limitations. Given the sampling approach applied, it is impossible to estimate a response rate and therefore to assume external validity. It is possible that those dentists that were most impacted by lockdown responded to our survey, or vice versa. This limits the generalisability of our data and is an acknowledged inherent weakness in our sampling approach. Another weakness arises from the potential for multiple interpretations of the term lockdown, depending on where respondents lived. Lastly, the data collected are based on self-report, a method selected in the absence of comparable international clinical datasets. It is acknowledged that respondents may produce biased data through recall bias and socially desirable responding, to name but two.

At a time when society is adjusting to a new normal, our data highlight the risk of an enduring, disproportionately negative impact on people with disabilities’ access to oral healthcare due to COVID-19. There is a need for oral healthcare and disability advocates to recognise this decline and to advocate not only for a return to pre-COVID services, but for radically improved access to care. The results of this study demonstrate positive changes in practice too, particularly the uptake of remote consultations. While not a replacement for traditional care, it has potential to be of particular advantage to people with disabilities in the future. Action in this regard will help address concerns surrounding the delivery of future care to meet routine treatment needs (30).

## Conclusions

During the first lockdown of the COVID-19 pandemic, there was a significant negative impact on the delivery of dental care to people with disabilities around the globe. Overall, oral healthcare access was significantly restricted with access to sedation and general anaesthesia particularly affected. While access improved somewhat as restrictions lifted, a negative impact persisted. A positive outcome may be the emergence of teledentistry as an option to address some of the traditional longstanding barriers to care for people with disabilities, which should be investigated and developed ongoing. As further lockdowns and restrictions arise, there is a need to protect access to emergency, restorative and preventive dental services for people with disabilities, so as to find balance in maintaining access to dental care and oral health in a way that minimises harm.
